# Physical modalities for the treatment of knee osteoarthritis: a network meta-analysis

**DOI:** 10.1007/s40520-025-03015-6

**Published:** 2025-04-07

**Authors:** Xiangzhou Lan, Lingjia Li, Qing Jia, Fangyi He, Gaoyan Kuang, Weike Zeng, Miao Chen, Cheng Guo, Zhi Wen, Qing Chen

**Affiliations:** 1 The First Affiliated Hospital of Hunan University of Traditional Chinese Medicine, Changsha, Hunan 410007 China; 2 School of Nursing, Hunan University of Chinese Medicine, Changsha, Hunan 410208 China; 3 Changsha Modern Nurse Magazine Co., LTD, Changsha, Hunan 410011 China

**Keywords:** Knee osteoarthritis, Physical modalities therapy, Network meta-analysis, Pain relief, Joint function

## Abstract

**Objective:**

This network meta-analysis (NMA) aimed to compare the efficacy of various physical modalities in alleviating pain, stiffness, and functional impairment in patients with knee osteoarthritis (KOA).

**Methods:**

In accordance with PRISMA-P guidelines, we systematically searched nine databases(CNKI, VIP Database, Wanfang Database, SinoMed, PubMed, Embase, CINAHL, Web of Science, and the Cochrane Library) from inception to October 2024 to identify randomized controlled trials (RCTs) evaluating physical therapies for KOA. The interventions assessed included electrical stimulation therapy (EST), low-level light therapy (LLLT), thermotherapy (TT), cryotherapy (CT), and extracorporeal shock wave therapy (ESWT), with resistance and range of motion exercises (RRE) serving as comparators. Outcome measures comprised the Visual Analog Scale (VAS), Western Ontario and McMaster Universities Osteoarthritis Index (WOMAC), and 6-minute walk test (6 MWT). Bayesian network meta-analyses and pairwise meta-analyses were performed using Stata 17.0 and R 4.4.1 software.

**Results:**

32 RCTs involving 2,078 participants were included. LLLT demonstrated the highest efficacy for pain reduction (VAS: MD=–3.32, 95% CI:–3.82 to–0.75; WOMAC pain: MD=–3.74, 95% CI:–6.68 to–0.72) and joint function improvement (SUCRA = 79.8). ESWT ranked second for pain relief (VAS: MD=–1.31, 95% CI:–2.42 to–0.16) and mobility enhancement (6 MWT: SUCRA = 71.5), while TT showed superior efficacy in reducing stiffness (WOMAC stiffness: MD=–2.09, 95%CI:–3.06 to–0.94; SUCRA = 98.1). In contrast, ultrasonic therapy (UT) did not provide significant benefits.

**Conclusions:**

The findings suggest that LLLT and ESWT may be optimal for pain relief and functional improvement in patients with KOA, whereas TT appears to be the most effective in reducing stiffness. Optimal dosing parameters of these physical modalities are crucial for maximizing clinical benefits. Clinicians should individualize treatment strategies based on patient-specific factors. Future large-scale RCTs are warranted to validate these protocols and address the heterogeneity of existing evidence.

**Clinical trial number:**

Not applicable.

**Supplementary Information:**

The online version contains supplementary material available at 10.1007/s40520-025-03015-6.

## Introduction

Knee osteoarthritis (KOA) is a degenerative joint disease characterized by progressive cartilage loss, remodeling of the subchondral bone, and synovial inflammation, and is one of the most common causes of disability in older adults. Its clinical features, which include chronic pain, stiffness, and diminished joint function, substantially impair quality of life [1–3]. Epidemiological studies indicate that approximately 13–15% of individuals aged ≥ 40 years are affected by KOA, with prevalence rates exceeding 30% among those aged ≥ 65 years, particularly in women [4, 5]. Given the aging global population, the burden of KOA is expected to increase, underscoring the urgent need for effective therapeutic strategies [6, 7].

Current management approaches for KOA primarily focus on symptom relief through pharmacological intervention. Nonsteroidal anti-inflammatory drugs (NSAIDs), corticosteroids, and analgesics are widely prescribed because of their rapid analgesic and anti-inflammatory effects [8–10]. However, these treatments do not modify the underlying disease progression and are associated with dose-dependent adverse effects such as gastrointestinal complications (e.g., ulcers), cardiovascular risks, and renal impairment, particularly with long-term use [11, 12]. These limitations have prompted interest in alternative methods, including minimally invasive and nonpharmacological techniques.

Minimally invasive therapies, such as intra-articular hyaluronic acid (HA) injections, platelet-rich plasma (PRP), and radiofrequency ablation, are designed to provide sustained symptom relief by directly addressing the joint pathology. Although HA injections are recommended by some clinical guidelines, their efficacy has been inconsistent, with meta-analyses reporting only modest improvements in pain and function compared to placebo [13, 14]. Similarly, PRP therapy has produced heterogeneous outcomes, likely owing to differences in preparation protocols and patient selection [15]. Furthermore, although radiofrequency ablation may attenuate nociceptive signaling, its invasive nature and associated risks, including infection and nerve damage, limit its broader adoption [16]. Cost-effectiveness analyses have also raised concerns regarding the long-term value of these interventions, especially in resource-limited settings [17].

In contrast, physical modalities offer noninvasive and cost-effective alternatives with favorable safety profiles. Techniques such as low-level laser therapy (LLLT), extracorporeal shockwave therapy (ESWT), and thermotherapy (TT) target pain pathways by modulating inflammatory cytokines, enhancing tissue perfusion, and promoting mechanotransduction-mediated cartilage repair [18, 19]. Clinical trials have suggested that these modalities can effectively improve pain, stiffness, and functional capacity in KOA [20, 21]. However, the comparative effectiveness of these physical therapies remains unclear because of heterogeneous study designs, variable treatment parameters, and lack of direct comparison trials [22, 23].

To address these gaps, this network meta-analysis (NMA) synthesized evidence from randomized controlled trials (RCTs) to evaluate the relative efficacy of various physical modalities in managing KOA. The analysis focused on validated endpoints: pain reduction as measured by the visual analog scale (VAS), functional improvement as assessed by the Western Ontario and McMaster Universities Osteoarthritis Index (WOMAC), and mobility as evaluated by the 6-minute walk test (6 MWT). By reconciling inconsistencies in the literature and providing hierarchical rankings of interventions, our findings aim to inform evidence-based clinical decision making and optimize therapeutic strategies for KOA.

## Methods

This study was conducted in accordance with the PRISMA-P guidelines for network meta-analyses [24]. The study protocol was prospectively registered in the PROSPERO database (registration number CRD42024601821).

### Literature search

A systematic search was conducted in nine Chinese and English databases, including the China National Knowledge Infrastructure (CNKI), VIP Chinese Science and Technology Journal Database, Wanfang Database, Chinese Biomedical Literature Service System (SinoMed), PubMed, Embase, CINAHL, Web of Science, and Cochrane Library. The search covered studies published from the inception of each database until October 20, 2024. The strategy combined MeSH terms with entry terms and employed keywords such as “Physical Therapy Modalities” “Osteoarthritis, Knee” “KOA” “Electric Stimulation Therapy” “Low-Level Light Therapy” and “Ultrasonic Therapy” among others. A representative search strategy for PubMed is provided in Table 1, and the complete search strings for all the databases are available in Supplementary Table S1.

**Table 1:** Search strategy (taking pubmed as an example)

Steps	Search terms
#1	“Physical Therapy Modalities“[MeSH Terms]
#2	“Physical Modality Therapy“[Title/Abstract] OR “Electric Stimulation Therapy“[Title/Abstract] OR “Low-Level Light Therapy“[Title/Abstract] OR “Ultrasonic Therapy“[Title/Abstract] OR “Cryotherapy“[Title/Abstract] OR “Hyperthermia, Induced“[Title/Abstract] OR “Extracorporeal Shockwave Therapy“[Title/Abstract] OR “Whole-Body Vibration” [Title/Abstract]
#3	#1 OR #2
#4	“Osteoarthritis, Knee” [MeSH Terms]
#5	“Knee Osteoarthritides“[Title/Abstract] OR “Knee Osteoarthritis“[Title/Abstract] OR “Osteoarthritis of the Knee“[Title/Abstract] OR “Osteoarthritis of Knee“[Title/Abstract] OR “Gonarthrosis“[Title/Abstract]
#6	#4 OR #5
#7	#3 AND #6

### Study selection

This review adhered to the PICOS framework to establish the eligibility criteria for the study. The studies included adult patients with a clinical diagnosis of knee osteoarthritis, with no restrictions on race, sex, age, or symptom duration. The intervention group received physical modality therapy, whereas the control group underwent routine rehabilitation exercises (RRE) or an alternative physical modality different from that used in the intervention group. Physical modalities examined included electrical stimulation therapy (EST), low-level light therapy (LLLT), ultrasonic therapy (UT), cryotherapy (CT), thermotherapy (TT), extracorporeal shock wave therapy (ESWT), and whole-body vibration therapy (WBVT) (Table 2). Outcomes were assessed using the VAS, WOMAC, and 6 MWT. Randomized controlled trials (RCTs) were included. Studies were excluded if they were duplicates, were published in languages other than Chinese or English, contained duplicate data, did not assess relevant outcome measures, had incomplete or unreported data, or lacked full-text availability.

**Table 2:** Overview of treatment groups

RRE	Routine Rehabilitation Exercises
EST	Electric Stimulation Therapy
LLLT	Low-Level Light Therapy
UT	Ultrasonic Therapy
CT	Cryotherapy
TT	Thermotherapy
ESWT	Extracorporeal Shock Wave Therapy
WBVT	Whole-Body Vibration Therapy
EST_UT	Combined use of Electric Stimulation Therapy and Ultrasonic Therapy

### Data extraction

The literature retrieved from the electronic searches was imported into Note Express (Version 4.0.0.9855) for management. Duplicate records were removed first, followed by title and abstract screening based on the predefined inclusion and exclusion criteria. Full-text reviews were conducted for the studies that met the eligibility criteria. Two researchers (Xiangzhou Lan and Lingjia Li) independently performed the screening, with discrepancies resolved by a third researcher (Weike Zeng). Following the final selection, the two researchers extracted relevant data, including the author, publication year, country, patient sample size, intervention duration (weeks), and outcome measures.

### Risk of bias within individual studies

Two reviewers (Qing Jia and Fangyi He) independently assessed the methodological quality of all included studies using the Revised Cochrane Risk of Bias Tool for Randomized Trials (RoB 2) [25]. This tool evaluates bias across five domains: (1) bias arising from the randomization process, (2) bias due to deviations from the intended interventions (effect of assignment to intervention), (3) bias due to missing outcome data, (4) bias in outcome measurement, and (5) bias in the selection of the reported result. Each domain was assessed using signaling questions to facilitate evaluation. The risk of bias for each domain was categorized as “Low risk” “Some concerns” or “High risk”.

### Statistical analysis

The meta-analysis was conducted using Stata 17.0 for pairwise comparisons, generating network and funnel plots [26]. Continuous data were analyzed using mean difference (MD) and 95% confidence interval (CI) as effect size metrics. When a closed loop was detected in the network plot, the node-splitting method was applied to assess inconsistency. A P-value > 0.05 indicated no significant inconsistency between direct and indirect comparisons, suggesting consistency. Bayesian network meta-analysis was performed using the BUGSnet package [27] in R 4.4.1, generating forest plots, league tables, and Surface Under the Cumulative Ranking Curve (SUCRA) plots. The Markov Chain Monte Carlo (MCMC) simulation included an adaptation period of 1000 iterations, a burn-in period of 1000 iterations, and 10,000 iterations. Sensitivity and subgroup analyses were performed as required.

## Results

### Literature search and quality assessment

**Fig. 1:**
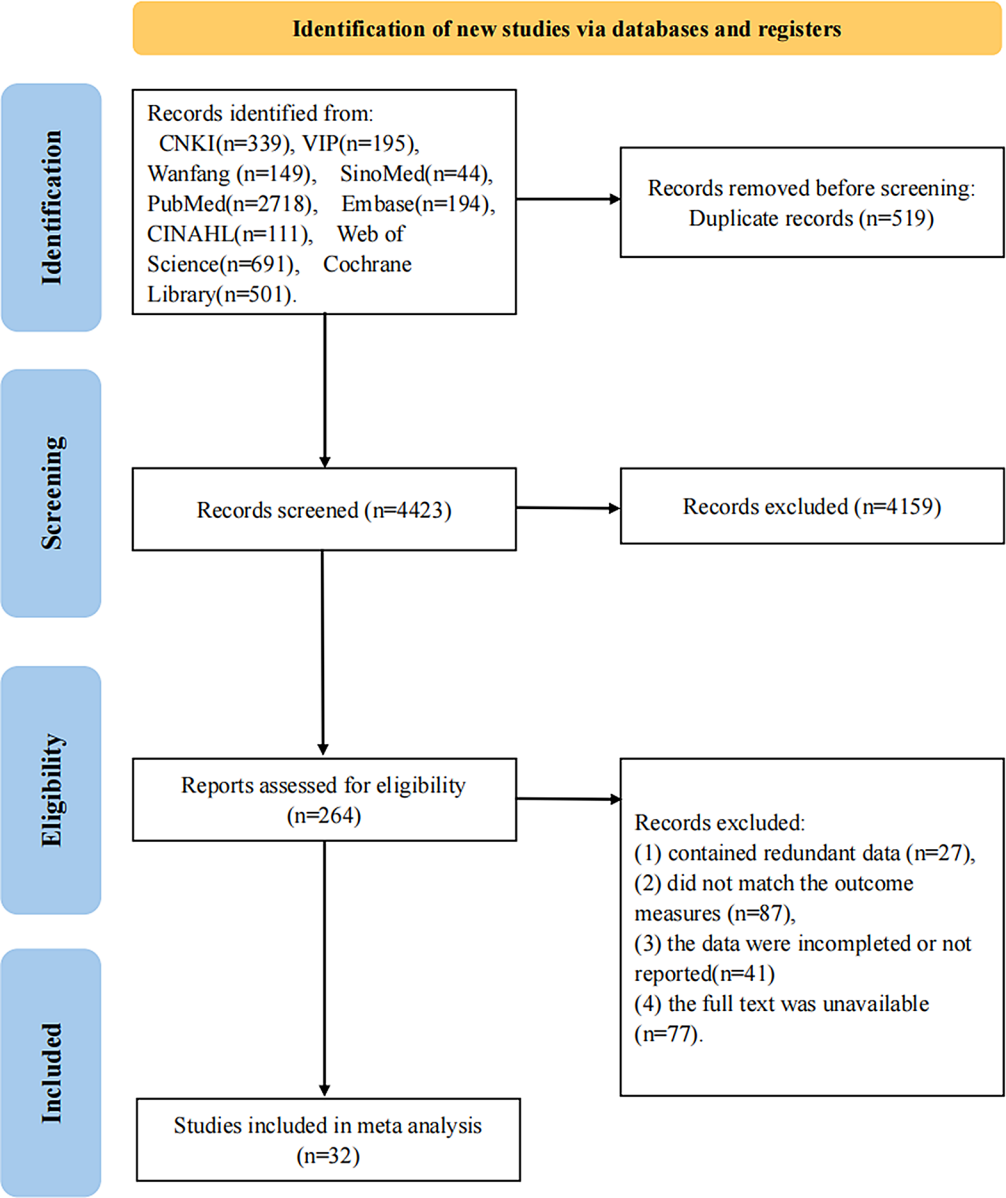
PRISMA flowchart for searching and selecting eligible studies

A total of 4,942 articles were initially identified from nine databases: CNKI (*n* = 339), VIP (*n* = 195), Wanfang (*n* = 149), SinoMed (*n* = 44), PubMed (*n* = 2,718), Embase (*n* = 194), CINAHL (*n* = 111), Web of Science (*n* = 691), and the Cochrane Library (*n* = 501). Following a stepwise screening process, 32 randomized controlled trials (RCTs) were included, comprising 2,078 participants with a mean age range of 55–72 years, of whom 67.5% were female. The sample size per study ranged from 18 patients to 240 patients. The literature screening process is presented in Fig. 1, and the basic characteristics of the included studies are summarized in Table 3.

**Fig. 2:**
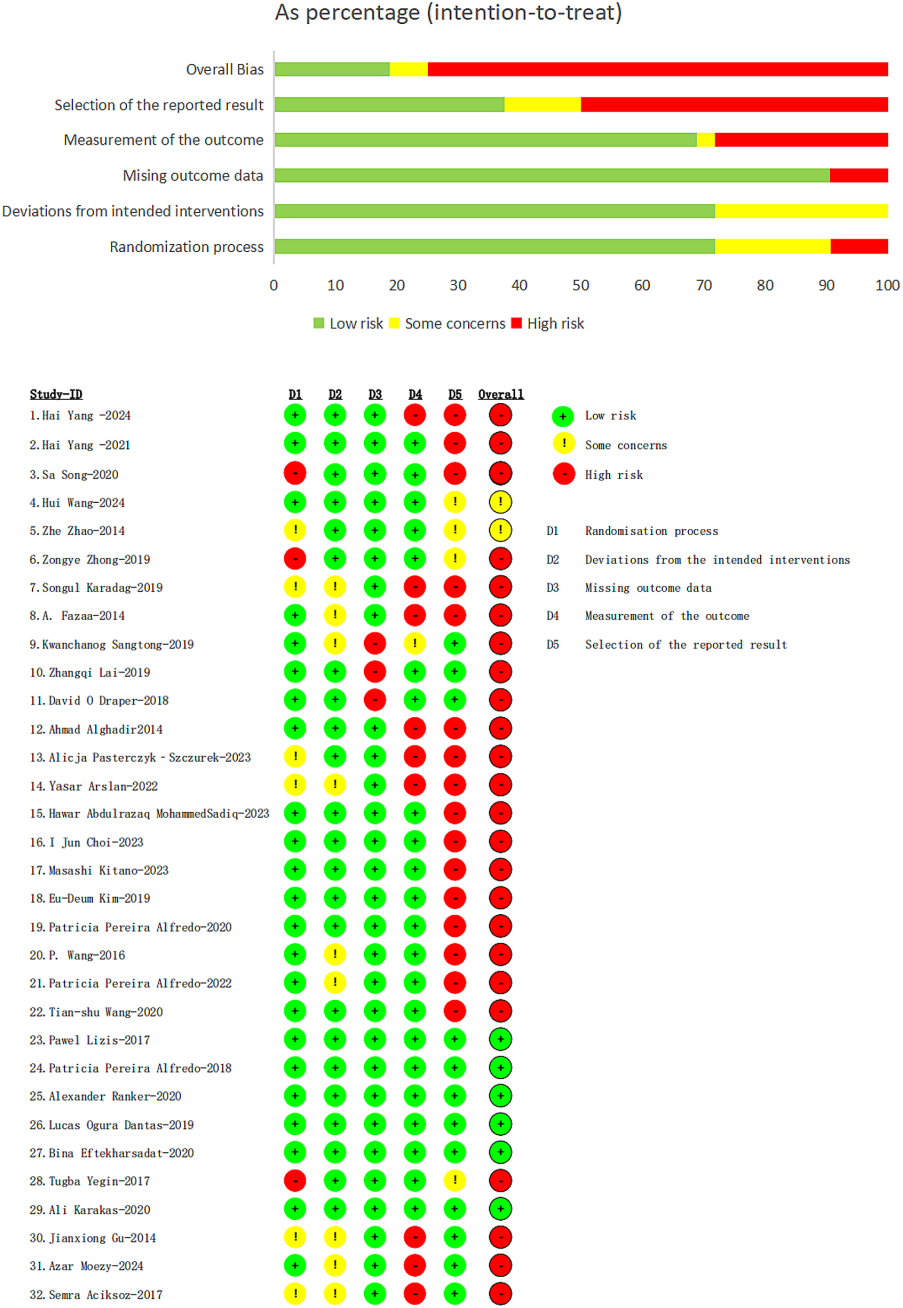
Risk of bias summary

Of the 32 included studies, six [50–54, 56] were assessed as having a low overall risk of bias, two [31, 32] were rated as raising some concerns, and the remaining studies were deemed to have a high risk of bias. In most cases, the high risk of bias was attributed to selective reporting by assessors. Three studies by Song et al. [30, 33, 55] exhibited a high risk of bias associated with the randomization process. Three studies [36–38] had a high risk of bias due to missing outcome data, whereas nine studies [28, 35, 36, 39–41, 57–59] had a high risk of bias related to outcome measurement, as shown in Fig. 2. Studies with three or more domains classified as having a high risk of bias were excluded from the analysis.

**Table 3:** Basic characteristics of the included studies

Authors	Country	Sample size		Gender (M/F)		Interventions		Treatment course (week)	Outcome measures
		T	C	T	C	T	C		
Hai Yang-2024[28]	China	61	61	13/48	22/39	EST_UT	UT	4	VAS, WOMAC,6MWT
Hai Yang-2021[29]	China	30	30	9/21	6/24	EST_UT	UT	2	VAS,6MWT
Sa Song-2020[30]	China	49	49	28/21	32/17	UT	RRE	8	VAS
Hui Wang-2024[31]	China	65	65	33/32	35/30	ESWT	RRE	8	VAS,6MWT
Zhe Zhao-2014[32]	China	34	36	14/20	11/25	ESWT	RRE	4	VAS
Zongye Zhong-2019[33]	China	32	31	11/21	12/19	ESWT	RRE	4	VAS, WOMAC
Songul Karadag-2019[34]	Turkey	15	17	2/13	2/15	TT	RRE	4	VAS, WOMAC
A. Fazaa-2014[35]	Tunisia	119	121	30/89	31/90	TT	RRE	3	VAS
Kwanchanog Sangtong-2019[36]	Thailand	74	74	5/69	8/66	EST_UT	UT	2	VAS
Zhangqi Lai-2019[37]	China	20	21	4/16	1/20	WBVT	RRE	8	6MWT
David O Draper-2018[38]	United States	51	33	23/28	16/17	UT	RRE	6	VAS
Ahmad Alghadir-2014[39]	Saudi Arabia	20	20	10/10	12/8	LLLT	RRE	4	VAS, WOMAC
Alicja Pasterczyk-Szczurek-2023[40]	Poland	16	16	1/15	4/12	WBVT	RRE	3	VAS
Yasar Arslan-2022[41]	Turkey	26	25	4/22	2/23	ESWT	RRE	3	VAS, WOMAC
Hawar Abdulrazaq MohammedSadiq-2023[42]	Iraq	18	16	5/13	3/13	CT	RRE	8	WOMAC
I Jun Choi-2023[43]	Korea	9	9	4/5	5/4	ESWT	RRE	3	VAS
Masashi Kitano-2023[44]	Japan	13	13	3/10	2/11	UT	RRE	5	VAS
Eu-Deum Kim-2019[45]	Korea	19	19	3/16	5/14	EST_UT	EST	8	VAS, WOMAC
Patricia Pereira Alfredo-2020[46]	Brazil	20	20	5/15	6/14	UT	RRE	8	VAS, WOMAC
P. Wang-2016[47]	China	19	20	9/10	7/13	WBVT	RRE	12	VAS, WOMAC,6MWT
Patricia Pereira Alfredo-2022[48]	Brazil	20	20	4/16	3/17	LLLT	RRE	8	VAS, WOMAC
Tian-shu Wang-2020[49]	China	36	36	24/12	21/15	ESWT	RRE	10	VAS, WOMAC
Pawel Lizis-2017[50]	Poland	20	20	7/13	9/11	ESWT	RRE	5	WOMAC
Patricia Pereira Alfredo-2018[51]	Brazil	20	20	-	-	LLLT	RRE	8	VAS, WOMAC
Alexander Ranker-2020[52]	Germany	14	13	5/9	3/10	EST	RRE	3	6MWT
Lucas Ogura Dantas-2019[53]	Brazil	30	30	15/15	15/15	CT	RRE	1	VAS
Bina Eftekharsadat-2020[54]	Iran	25	25	0/25	3/22	ESWT	RRE	3	VAS, WOMAC
Tugba Yegin-2017[55]	Turkey	30	32	-	-	UT	RRE	2	6MWT
Ali Karakas-2020[56]	Germany	39	36	8/31	4/32	UT	RRE	8	VAS, WOMAC
Jianxiong Gu-2014[57]	China	30	30	12/18	11/19	LLLT	RRE	3	VAS
Azar Moezy-2024[58]	Iran	25	25	0/25	0/25	EST	RRE	4	VAS, WOMAC,6MWT
Semra Aciksoz-2017[59]	Turkey	32, 32, 32		5/27, 6/26, 7/25		TT, CT, RRE		3	VAS, WOMAC

*Note* M( Male); F( Female); T(Treatment group); C(Control group)

### Network relationships and consistency analysis

**Fig. 3:**
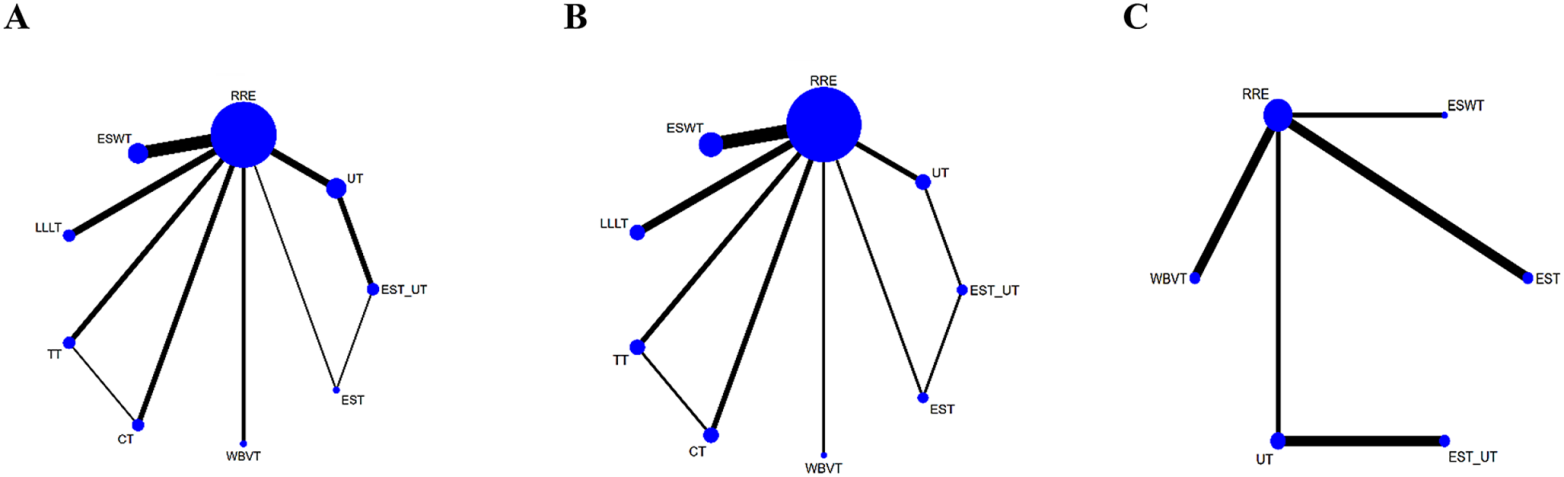
**A**-**C** Network Plots of the Visual Analogue Scale, Western Ontario and McMaster Universities Arthritis Index, and Six-Minute Walk Test

The network diagrams for the three outcome measures are shown in Fig. 3A-C. Twenty-nine studies reported VAS scores involving nine physical therapeutic modalities and routine rehabilitation exercises, forming two closed loops. Seventeen studies reported WOMAC scores, involving 9 physical therapeutic modalities and routine rehabilitation exercises, also forming two closed loops. Eight studies reported the 6 MWT, involving six physical therapeutic modalities and routine rehabilitation exercises, with no closed loops formed.

A global inconsistency test was conducted across all studies, revealing that the inconsistency for all three outcome measures was not significant (*P* > 0.05), indicating minimal inconsistency across the studies. Therefore, the consistency model was considered appropriate for the analysis. Additionally, a local inconsistency test was performed using the node-splitting method to compare direct and indirect comparisons within the network. The results showed no statistically significant inconsistencies, thus further supporting the validity of the consistency model.

### Visual analogue scale

**Fig. 4:**
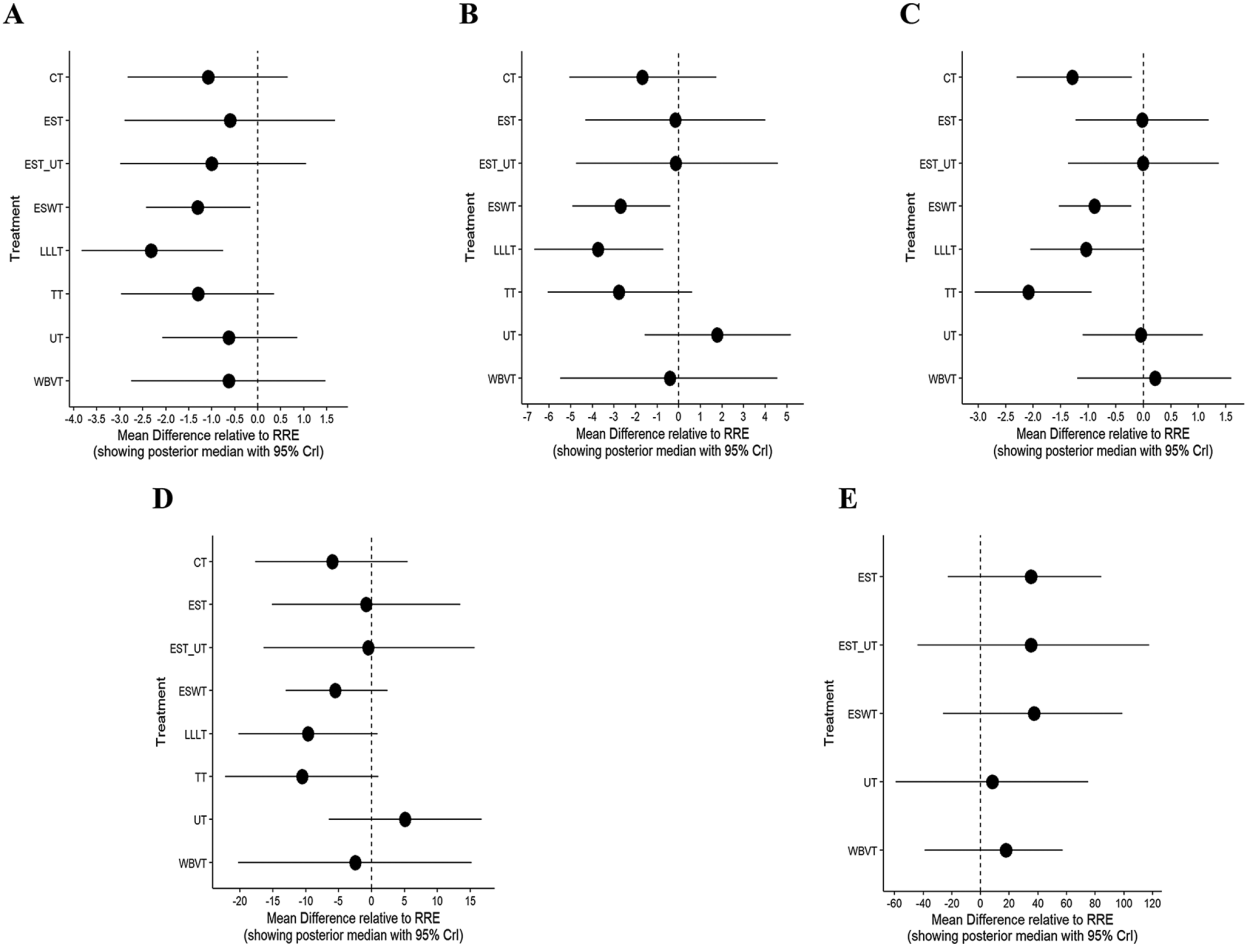
**A**-**E** Forest Plots of the Visual Analogue Scale, WOMAC Pain Subscale, WOMAC Stiffness Subscale, WOMAC Function Subscale, and Six-Minute Walk Test

**Fig. 5:**
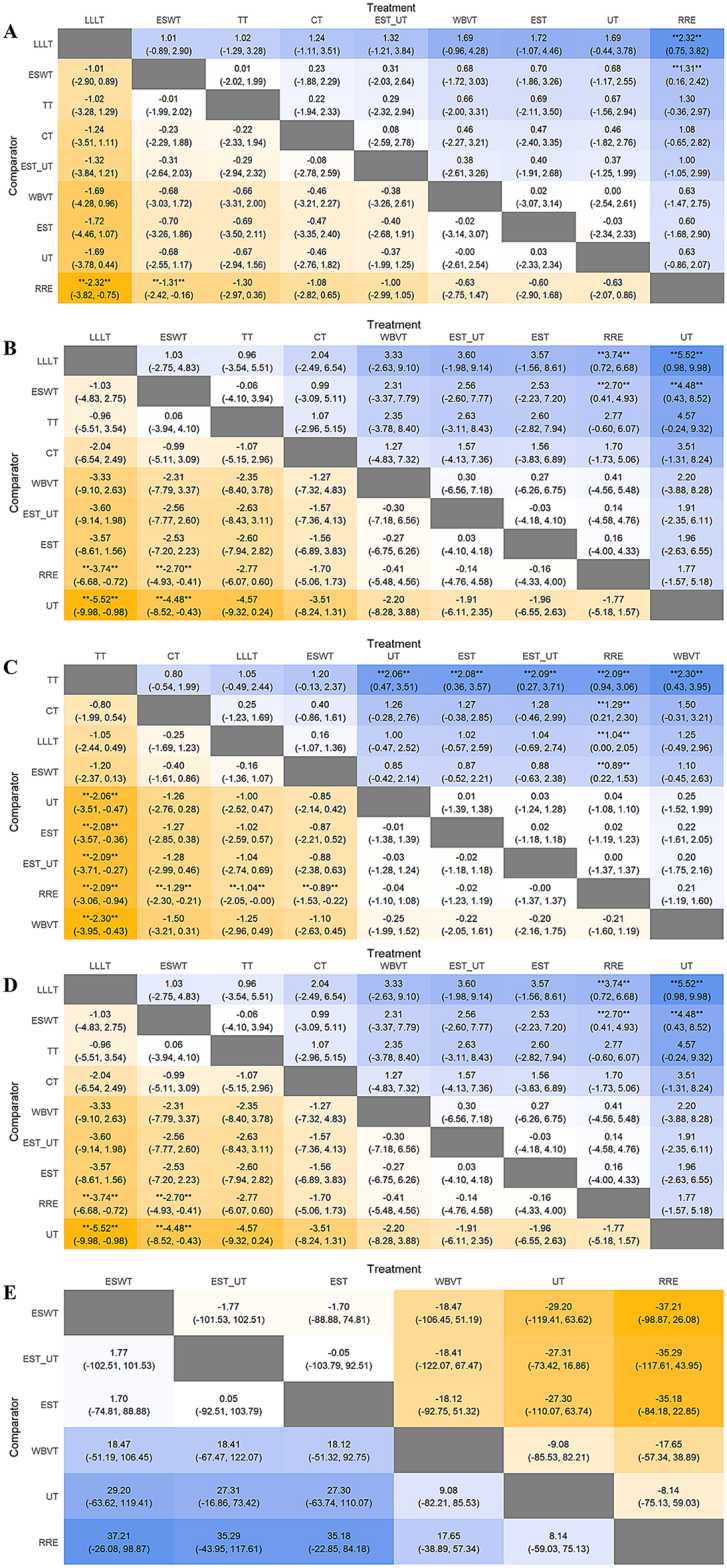
**A**-**E** League Tables of the Visual Analogue Scale, WOMAC Pain Subscale, WOMAC Stiffness Subscale, WOMAC Function Subscale, and Six-Minute Walk Test. Comparison of all treatment in the network, combined indirect and direct if, available. ** indicates that the difference is statistically significant

**Fig. 6:**
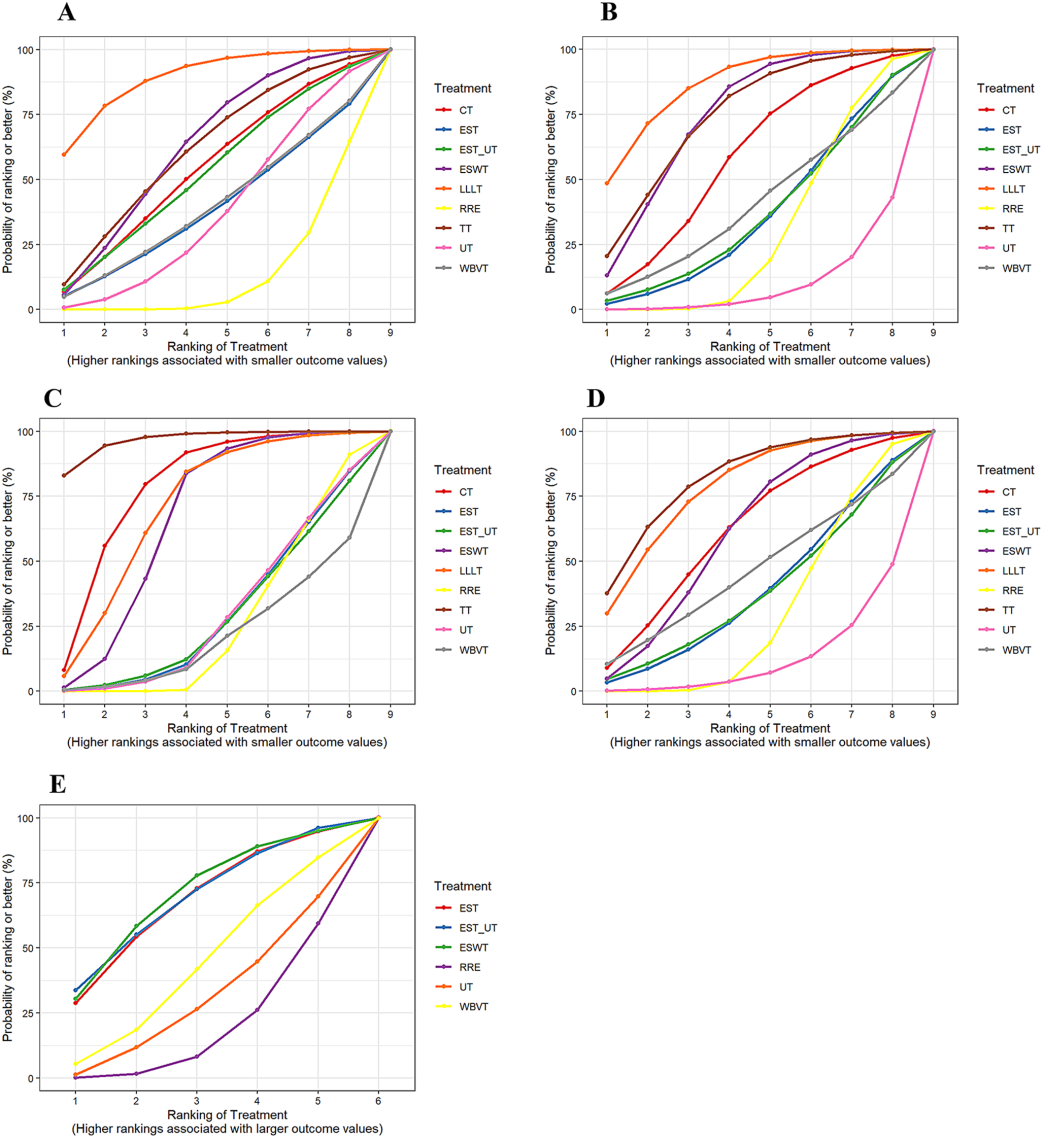
**A**-**E** SUCRA Plots of the Visual Analogue Scale, WOMAC Pain Subscale, WOMAC Stiffness Subscale, WOMAC Function Subscale, and Six-Minute Walk Test

A total of 27 studies involving nine interventions were included in the analysis of VAS scores. A forest plot with the RRE as the comparator is shown in Fig. 4A. All interventions resulted in a reduction in VAS scores compared to RRE. However, only LLLT (MD = -3.32, 95% CI = -3.82, -0.75) and ESWT (MD = -1.31, 95% CI = -2.42, -0.16) showed statistically significant differences compared with RRE (*P* < 0.05). These findings are depicted in the league and SUCRA plots (Figs. 5A and 6A). The SUCRA rankings were as follows: LLLT (90.1) > ESWT (62.8) > TT (61.9) > CT (54.4) > EST_UT (53.2) > WBVT (38.5) > EST (39.3) > UT (37.5) > RRE (12.4).

### Western Ontario and Mcmaster universities arthritis index

A total of 17 studies evaluated using the WOMAC scale, involving 9 interventions.

#### Pain subscale

A forest plot with the RRE as the comparator is shown in Fig. 4B. Except for UT, all interventions resulted in a reduction in the WOMAC pain subscale compared to RRE. However, only LLLT (MD = -3.74, 95% CI = -6.68, -0.72) and ESWT (MD = -2.70, 95% CI = -4.93, -0.41) demonstrated statistically significant differences compared with RRE (*P* < 0.05). These results are further illustrated in league and SUCRA plots (Figs. 5B and 6B, respectively). The SUCRA rankings were LLLT (88.5) > TT (77.0) > ESWT (75.7) > CT (59.2) > WBVT (39.9) > EST_UT (36.6) > EST (35.7) > RRE (29.7) > UT (7.8).

#### Stiffness subscale

The forest plot (Fig. 4C) revealed that WBVT was less effective than RRE in improving stiffness associated with knee osteoarthritis, whereas UT, EST, and EST/UT showed comparable effects to RRE. Compared to RRE, TT (MD = -2.09, 95% CI = -3.06 to -0.94) and CT (MD = -1.29, 95% CI = -2.30 to -0.21) demonstrated greater reductions in the WOMAC stiffness subscale, followed by LLLT (MD = -1.04, 95% CI = -2.05 to 0.00) and ESWT (MD = -0.89, 95% CI = -1.53 to -0.22). All the differences were statistically significant (*P* < 0.05). These findings were further visualized in league and SUCRA plots (Figs. 5C and 6C). SUCRA rankings were TT (98.1), CT (79.8), LLLT (71.8), ESWT (67.3), UT (29.1), EST (29.0), EST_UT (28.4), RRE (26.1), and WBVT (20.5).

#### Function subscale

A Forest plot with the RRE as the comparator is shown in Fig. 4D. Except for UT, all interventions resulted in a reduction in the WOMAC Function Subscale compared with RRE. However, none of the interventions showed significant differences compared to RRE. These results are further illustrated in league and SUCRA plots (Figs. 5D and 6D). The SUCRA rankings were TT (84.0) > LLLT (79.8) > CT (63.7) > ESWT (61.8) > WBVT (45.2) > EST (38.3) > EST_UT (38.0) > RRE (29.0) > UT (10.1).

### Six-minute walk test

Eight studies involving six interventions reported the outcomes of the 6 MWT. As shown in the forest plots (Fig. 4E), all interventions improved the 6-minute walking distance compared to RRE; however, the differences were not statistically significant. These results are further illustrated in league and SUCRA plots (Figs. 5E and 6E). The SUCRA ranking was as follows: ESWT (71.5); EST_UT (69.8); EST (68.2); WBVT (42.4); UT (29.4) and RRE (18.3).

### Publication bias

**Fig. 7:**
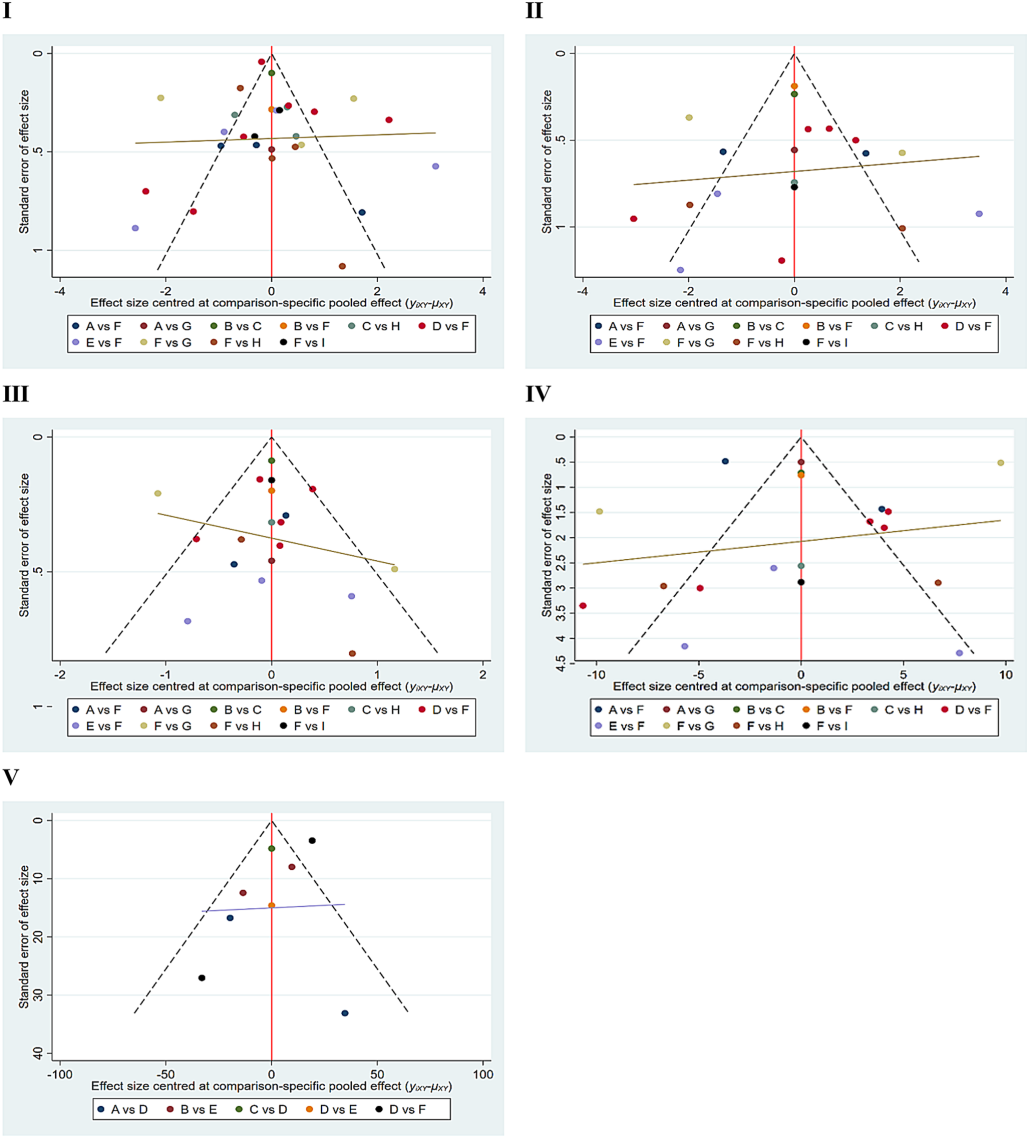
**I-V** Funnel plots of the Visual Analogue Scale, WOMAC Pain Subscale, WOMAC Stiffness Subscale, WOMAC Function Subscale, and Six-Minute Walk Test. The X-axis represents the effect size, while the Y-axis represents the standard error. In Fig I-IV, A: CT, B: EST, C: EST_UT, D: ESWT, E: LLLT, F: RRE, G: TT, H: UT, I: WBVT. In Fig V, A: EST; B: EST_UT; C: ESWT; D: RRE; E: UT; F: WBVT

Funnel plots were constructed for VAS scores, WOMAC Pain Subscale, the WOMAC Stiffness Subscale, the WOMAC Function Subscale, and 6WMT. The results indicated that The funnel plots for all five outcomes were approximately symmetric on both sides, suggesting a low probability of publication bias, as shown in Fig. 7 I-V.

## Discussion

In the present analysis, regarding the efficacy of pain relief in patients with KOA, both the VAS and WOMAC pain subscale scores indicated that LLLT, ESWT, and TT were among the top three modalities in the SUCRA analysis, with LLLT emerging as the most effective. LLLT has been shown to significantly alleviate pain and other adverse symptoms associated with KOA, a finding consistent with that of Oliveira et al. [60]. One study [61] demonstrated that LLLT improves local blood circulation, promotes tissue regeneration, and reduces pain and edema. Lou et al. [62] suggested that LLLT might inhibit the release of pro-inflammatory cytokines, thereby attenuating the inflammatory response, which plays a pivotal role in relieving pain and enhancing joint function. Additionally, a study [63] found that LLLT can reduce chondrocyte apoptosis, stimulate the synthesis of extracellular matrix (ECM) components, and decrease the release of inflammatory mediators and proteolytic enzymes in chronic joints, thu s, preventing degenerative changes in the joints and surrounding tissues, alleviating oxidative stress, and ultimately mitigating pain during the progression of KOA. ESWT ranked second in the SUCRA analysis, and its efficacy in relieving pain in patients with KOA has been substantiated by numerous randomized controlled trials [33, 64, 65]. The analgesic mechanisms of ESWT are likely multifactorial, involving anti-inflammatory effects [66], promotion of angiogenesis [67], reduction of joint effusion [43], and modulation of substance P release [68]. Recent studies [69] have indicated a negative linear relationship between ESWT dosage and clinical outcomes, suggesting that higher energy levels may result in greater clinical improvement in KOA treatment than lower energy settings. TT ranked third in the SUCRA analysis, although the difference compared with RRE was not statistically significant. It is well established that thermotherapy promotes muscle relaxation and enhances blood circulation at The affected site, thereby reducing pain and stiffness [70]. Although thermotherapy has demonstrated positive effects in KOA treatment, its use as a stand-alone intervention remains relatively limited. More frequently, it is combined with other modalities, such as electrotherapy [71] or moxibustion [72]. This study found that LLLT was ranked higher than ESWT, although the difference was not statistically significant. LLLT has been shown to have a significant effect on pain relief and disability reduction at specific dosages; however, the overall effect remains debatable, largely because of the influence of varying treatment parameters [73]. Notably, one study [74] reported that LLLT with wavelengths in the range of 904– The 905 nm laser is particularly effective in alleviating pain in patients with KOA. In contrast, while ESWT provides short-term pain relief, it is also associated with the potential for pain rebound [75]. Consequently, clinicians should adopt a comprehensive approach when selecting treatment options for KOA, carefully considering individual patient characteristics and preferences, to optimize therapeutic outcomes.

Second, with regard to the alleviation of stiffness in KOA patients, the results of this study indicated that TT ranked first in the SUCRA analysis, with a statistically significant difference compared with RRE (*P* < 0.05). A study by Gao et al. [76], using an animal model, found that thermotherapy could reduce congestion, edema in the joint capsule and synovial tissues, and cartilage surface wear, thereby alleviating joint stiffness. The underlying mechanism appears to be related to suppression of inflammatory factors. Another study [77] suggested that thermotherapy accelerated blood circulation and enhanced muscle and soft tissue metabolism, which contributed to the reduction in joint stiffness. These mechanisms may work synergistically by inhibiting inflammatory mediators, promoting local blood circulation, and enhancing tissue metabolism, thereby reducing knee joint stiffness [78, 79]. Consequently, thermotherapy may be an effective method to alleviate stiffness in patients with KOA. However, there is currently no evidence to suggest that thermotherapy has a distinct advantage over other physical modalities in reducing joint stiffness in patients with KOA, and large-scale randomized controlled trials are required. Furthermore, thermotherapy offers advantages, such as ease of administration, cost-effectiveness, and safety, and can be performed both at home and in other settings. However, caution is required when applying thermotherapy to patients with sensory impairments or poor local skin conditions as there is an increased risk of burns. Therefore, clinicians should carefully tailor interventions and dosages based on an individual patient’s condition to optimize the therapeutic outcomes. Moreover, with respect to the improvement of joint function in KOA patients, the results of this study demonstrated that LLLT and ESWT were the most effective modalities in the SUCRA ranking for the WOMAC Functional Subscale and 6 MWT, respectively. A study [80] by indicated that LLLT may help treat KOA by promoting tissue regeneration, thereby improving symptoms such as joint pain, stiffness, restricted joint mobility, and decreased walking function. Alves et al. [81] further suggested that LLLT can modulate the expression of IL-6 mRNA and reduce the number of neutrophils during the initial inflammatory phase, thereby alleviating the signs and symptoms of osteoarthritis. These findings suggest that the precise mechanism by which LLLT improves joint function in patients with KOA remains unclear and warrants further investigation in future studies. Similarly, numerous studies [82–84]have confirmed the effectiveness of ESWT in improving knee joint function. However, its exact mechanism of action remains unclear. One hypothesis proposed by researchers [85] posits that “ESWT may enhance the proliferation of target cells seeded on bioactive scaffolds, promote chondrogenic differentiation, and stimulate the production of extracellular matrix (ECM) in cartilage.” This hypothesis is based on the mechanical signaling transmitted by cells, where cells convert mechanical signals from shock waves into biochemical responses through integrins, ion channels, the cytoskeleton, growth factor receptors, and the nucleus. Additionally, by regulating gene expression and upregulating the release of various growth factors in the three-dimensional cartilage environment, ESWT has the potential to support cells (such as chondrocytes and stem cells) in simulating their optimal functions. Other studies [67, 86] have also shown that ESWT can promote the repair of cartilage and subchondral bone damage through the involvement of multiple cytokines, chemokines, proteins, and miRNAs. Although the efficacy of ESWT is well established, its underlying mechanisms require further investigation. In summary, both LLLT and ESWT may alleviate joint functional issues in KOA patients through distinct mechanisms. Healthcare professionals should consider the specific clinical conditions of patients when selecting the most appropriate physical treatment modality. In conclusion, regarding the improvement of pain and joint function in patients with KOA, both LLLT and ESWT demonstrated superior efficacy compared to other physical modalities. In terms of alleviating stiffness, TT showed greater advantages than the other treatments did. However, this study has several limitations: (1) only Chinese and English-language literature were included; (2) the 32 studies included in the analysis exhibited a relatively high risk of bias, with considerable variation in the frequency, duration, and protocols of the interventions, which may have affected the accuracy of the ranking results; and (3) because of the limited number of reports, the inclusion of stiffness and joint function indicators in the interventions was relatively sparse, which may introduce potential bias in the findings.

## Conclusion

This study compared the efficacy of nine physical modalities for the treatment of KOA. The results showed that LLLT and ESWT were superior to RRE, with LLLT being the most effective treatment. The TT appears to be the most effective modality for alleviating stiffness. LLLT has been found to be the most promising treatment for improving joint function. Regarding joint mobility, ESWT was more effective than other interventions. Different dosages of the physical modalities are crucial for their use and efficacy. Therefore, when interpreting the findings of this study, clinicians should consider factors such as patient condition, preferences, economic situation, and available facilities to select the most appropriate physical modality. Future research should involve larger randomized controlled trials to verify the efficacy of certain physical modalities or to explore the differences in the effectiveness of various physical interventions for KOA, with the aim of providing optimal treatment strategies and enhancing patient quality of life.

## Electronic supplementary material

Below is the link to the electronic supplementary material.

Supplementary Material 1

Supplementary Material 2

## Data Availability

No datasets were generated or analysed during the current study.
