# Supplementary material for: Physical modalities for the treatment of knee osteoarthritis: a network meta-analysis
Source: Aging Clin Exp Res. 2025 Apr 7;37(1):121. doi: 10.1007/s40520-025-03015-6 (PMC11976336; doi:10.1007/s40520-025-03015-6)
Supplement: Supplementary file 2 — Supplementary Material 2 [file 40520_2025_3015_MOESM2_ESM.docx]

Network Meta-Analysis of Physical Modality Therapy for Knee Osteoarthritis

**P（Participants）：**Adult patients clinically diagnosed with knee osteoarthritis (KOA), with no restrictions on race, gender, age, or symptom duration；

**I（Intervention）：**Physical Modality Therapy（Electric Stimulation Therapy OR Low-Level Light Therapy OR Ultrasonic Therapy OR Cryotherapy OR Hyperthermia, Induced OR Extracorporeal Shockwave Therapy OR Whole-Body Vibration）；

**C（Comparison）：**Resistance and Range of Motion Exercises (RRE) or physical modality therapies distinct from those administered to the intervention group；

**O（Outcomes）：**Outcomes were measured by VAS, the WOMAC and the 6MWT；

**S（Study design）：**RCTs。

The databases searched include: CNKI, Wanfang, VIP, SinoMed, PubMed, Web of Science (WOS), CINAHL, Embase, and the Cochrane Library. The search period covers from the inception of the databases to October 15, 2024.

1. **Electric Stimulation Therapy，EST**

MeSH：**Electric Stimulation Therapy**

Entry Terms：Therapy, Electric Stimulation/Stimulation Therapy, Electric/Electrotherapy/Therapeutic Electric Stimulation/Electric Stimulation, Therapeutic/Stimulation, Therapeutic Electric/Electrical Stimulation Therapy/Stimulation Therapy, Electrical/Therapy, Electrical Stimulation/Therapeutic Electrical Stimulation/Electrical Stimulation, Therapeutic/Stimulation, Therapeutic Electrical/Interferential Current Electrotherapy/Electrotherapy, Interferential Current

1. **Low-Level Light Therapy,LLLT**

MeSH：Low-Level Light Therapy

Entry Terms：Light Therapies, Low-Level/Light Therapy, Low-Level/Low-Level Light Therapies/Low Level Light Therapy/Therapies, Low-Level Light/Therapy, Low-Level Light/LLLT/Photobiomodulation Therapy/Photobiomodulation Therapies/Therapies, Photobiomodulation/Therapy, Photobiomodulation/Photobiomodulation/Photobiomodulations/Laser Therapy, Low-Level/Laser Therapies, Low-Level/Laser Therapy, Low Level/Low-Level Laser Therapies/Laser Biostimulation/Biostimulation, Laser/Laser Irradiation, Low-Power/Irradiation, Low-Power Laser/Laser Irradiation, Low Power/Laser Phototherapy/Phototherapy, Laser/Laser Therapy, Low-Power/Laser Therapies, Low-Power/Laser Therapy, Low Power/Low-Power Laser Therapies/Low-Level Laser Therapy/Low Level Laser Therapy/Low-Power Laser Irradiation/Low Power Laser Irradiation/Low-Power Laser Therapy/Low Power Laser Therapy

1. **Ultrasonic Therapy,UT**

MeSH：Ultrasonic Therapy

Entry Terms：Therapies, Ultrasonic/Ultrasonic Therapies/Ultrasound Therapy/Therapies, Ultrasound/Therapy, Ultrasound/Ultrasound Therapies/Therapeutic Ultrasound/Ultrasound, Therapeutic/Therapy, Ultrasonic

1. **Cryotherapy,CT**

MeSH：Cryotherapy

Entry Terms：Cryotherapies/Cold Therapy/Cold Therapies/Therapies, Cold/Therapy, Cold

1. **Thermotherapy,TT**

MeSH：Hyperthermia, Induced

Entry Terms：Thermotherapy/Therapy, Fever/Fever Therapy/Induced Hyperthermia/Therapeutic Hyperthermia/Hyperthermia, Therapeutic/Hyperthermia, Local/Local Hyperthermia

1. **Extracorporeal Shock Wave Therapy, ESWT**

MeSH：Extracorporeal Shockwave Therapy

Entry Terms：Extracorporeal Shockwave Therapies/Shockwave Therapies, Extracorporeal/Shockwave Therapy, Extracorporeal/Therapy, Extracorporeal Shockwave/Extracorporeal Shock Wave Therapy/Shock Wave Therapy/Shock Wave Therapies/Therapy, Shock Wave/Extracorporeal High-Intensity Focused Ultrasound Therapy/Extracorporeal High Intensity Focused Ultrasound Therapy/High-Intensity Focused Ultrasound Therapy/High Intensity Focused Ultrasound Therapy/HIFU Therapy/HIFU Therapies/Therapy, HIFU

1. **Whole-Body Vibration Therapy, WBVT**

MeSH：Whole Body Vibration

Entry Terms：No items found.

1. **Knee Osteoarthritides，KOA**

MeSH：Osteoarthritis,Knee

Entry Terms：Knee Osteoarthritides/Knee Osteoarthritis/Osteoarthritis of the Knee/Osteoarthritis of Knee

**（1）CNKI：339**

SU=‘物理因子’+‘物理’+‘电疗’+‘激光’+‘超声波’+‘冷疗’+‘热疗’+‘冲击波’+‘震动’ AND SU=‘膝骨关节炎’+‘KOA’+‘膝骨性关节炎’+‘膝关节炎’

**（2）Wanfang：149**

题名或关键词:(“物理因子”or“物理”or“电疗”or“激光”or“超声波”or“冷疗”or“热疗”or“冲击波”or“震动”) and 题名或关键词:(“膝骨关节炎”or“KOA”or“膝骨性关节炎”or“膝关节炎”)

**（3）VIP：195**

M=(物理因子 OR 物理 OR 电疗 OR 激光 OR 超声波 OR 冷疗 OR 热疗 OR 冲击波 OR 震动) AND M=(膝骨关节炎 OR KOA OR膝骨性关节炎 OR膝关节炎)

**（4）SinoMed：44**

( "物理因子"[标题:智能] OR "物理"[标题:智能] OR "电疗"[标题:智能] OR "激光"[标题:智能] OR "超声波"[标题:智能] OR "冷疗"[标题:智能] OR "热疗"[标题:智能] OR "冲击波"[标题:智能] OR "震动"[标题:智能]) AND( "膝骨关节炎"[标题:智能] OR "KOA"[标题:智能] OR "膝骨性关节炎"[标题:智能] OR "膝关节炎"[标题:智能])

**（5）PubMed：2718**

#1 "Physical Therapy Modalities"[MeSH Terms]

#2 "Physical Modality Therapy"[Title/Abstract] OR "Electric Stimulation Therapy"[Title/Abstract] OR "Low-Level Light Therapy"[Title/Abstract] OR "Ultrasonic Therapy"[Title/Abstract] OR "Cryotherapy"[Title/Abstract] OR "Hyperthermia, Induced"[Title/Abstract] OR "Extracorporeal Shockwave Therapy"[Title/Abstract] OR "Whole-Body Vibration" [Title/Abstract]

#3 #1 OR #2

#4 "Osteoarthritis, Knee" [MeSH Terms]

#5 "Knee Osteoarthritides"[Title/Abstract] OR "Knee Osteoarthritis"[Title/Abstract] OR "Osteoarthritis of the Knee"[Title/Abstract] OR "Osteoarthritis of Knee"[Title/Abstract] OR "Gonarthrosis"[Title/Abstract]

#6 #4 OR #5

#7 #3 AND #6

**（6）WOS：691**

((TS=( "Physical Therapy Modalities")) OR TS=( "Physical Modality Therapy" OR "Electric Stimulation Therapy" OR "Low-Level Light Therapy" OR "Ultrasonic Therapy" OR "Cryotherapy" OR "Hyperthermia, Induced" OR "Extracorporeal Shockwave Therapy" OR "Whole-Body Vibration")) AND TI=("Osteoarthritis, Knee" OR "Knee Osteoarthritides" OR "Knee Osteoarthritis" OR "Osteoarthritis of the Knee" OR "Osteoarthritis of Knee")

**（7）CINAHL：111**

SU ( ("Osteoarthritis, Knee" OR "Knee Osteoarthritides" OR "Knee Osteoarthritis" OR "Osteoarthritis of the Knee" OR "Osteoarthritis of Knee") ) AND SU ( ("Physical Modality Therapy" OR "Physical Therapy Modalities" OR "Electric Stimulation Therapy" OR "Low-Level Light Therapy" OR "Ultrasonic Therapy" OR "Cryotherapy" OR "Hyperthermia, Induced" OR "Extracorporeal Shockwave Therapy" OR "Whole-Body Vibration") )

**（8）Embase：194**

('osteoarthritis, knee':ab,ti OR 'knee osteoarthritides':ab,ti OR 'knee osteoarthritis':ab,ti OR 'osteoarthritis of the knee':ab,ti OR 'osteoarthritis of knee':ab,ti) AND ('physical modality therapy':ab,ti OR 'physical therapy modalities':ab,ti OR 'electric stimulation therapy':ab,ti OR 'low-level light therapy':ab,ti OR 'ultrasonic therapy':ab,ti OR cryotherapy:ab,ti OR 'hyperthermia, induced':ab,ti OR 'extracorporeal shockwave therapy':ab,ti OR 'whole-body vibration':ab,ti)

**（9）Cochrane Library：501**

#1 ("Osteoarthritis, Knee" OR "Knee Osteoarthritides" OR "Knee Osteoarthritis" OR "Osteoarthritis of the Knee" OR "Osteoarthritis of Knee") :ti,ab,tw

#2 ("Physical Modality Therapy" OR "Physical Therapy Modalities" OR "Electric Stimulation Therapy" OR "Low-Level Light Therapy" OR "Ultrasonic Therapy" OR "Cryotherapy" OR "Hyperthermia, Induced" OR "Extracorporeal Shockwave Therapy" OR "Whole-Body Vibration"):ti,ab,tw

#3 #1 AND #2

A total of 4942 articles were retrieved from 9 databases.

(1) Removal weight: 519, remaining 4423.

(2)Exclusion:

① Non-KOA patients: 7+12+1+14+25+332=391

② Non-physical factor therapy: 139+108+31+90+60+993=1079

③ Non-RCTs: 21+12+1+74+40+1192=1340

④ Too long ago: 83+39+61+13+908+148+27+20+50=1349

Remaining: 78+7+16+4+53+50+10+18+28=264 articles.

(3) After reading the full text, exclusion:

① data duplication: 27

② outcome indicators inconsistent: 87

③ data incomplete or unreported: 41

④ Full text unavailable: 77

(4) Final inclusion: 32.
